# Inhibition of acid sphingomyelinase increases regulatory T cells in humans

**DOI:** 10.1093/braincomms/fcab020

**Published:** 2021-03-05

**Authors:** Teresa Wiese, Fabio Dennstädt, Claudia Hollmann, Saskia Stonawski, Catherina Wurst, Julian Fink, Erika Gorte, Putri Mandasari, Katharina Domschke, Leif Hommers, Bernard Vanhove, Fabian Schumacher, Burkhard Kleuser, Jürgen Seibel, Jan Rohr, Mathias Buttmann, Andreas Menke, Jürgen Schneider-Schaulies, Niklas Beyersdorf

**Affiliations:** 1 Institute for Virology and Immunobiology, University of Würzburg, Würzburg 97078, Germany; 2 Department of Psychiatry, Psychosomatics and Psychotherapy, Center of Mental Health, University Hospital of Würzburg, Würzburg 97080, Germany; 3 Institute of Organic Chemistry, University of Würzburg, Würzburg 97074, Germany; 4 Department of Psychiatry and Psychotherapy, Medical Center—University of Freiburg, Faculty of Medicine, University of Freiburg, Freiburg 79104, Germany; 5 Comprehensive Heart Failure Center, University Hospital of Würzburg, Würzburg 97080, Germany; 6 Interdisciplinary Center for Clinical Research, University of Würzburg, Würzburg 97080, Germany; 7 Centre de Recherche en Transplantation et Immunologie UMR 1064, INSERM, Université de Nantes, Nantes, France; 8 Institut de Transplantation Urologie Néphrologie (ITUN), CHU Nantes, Nantes, France; 9 OSE Immunotherapeutics S.A., Nantes, France; 10 Institute of Nutritional Science, University of Potsdam, Nuthetal D-14558, Germany; 11 Center for Chronic Immunodeficiency (CCI), Medical Center—University of Freiburg, Freiburg 79106, Germany; 12 Department of Neurology, Caritas Hospital, Bad Mergentheim 97980, Germany; 13 Department of Neurology, University Hospital Würzburg, Würzburg 97080, Germany; 14 Medical Park Chiemseeblick, Bernau-Felden 83233, Germany

**Keywords:** acid sphingomyelinase, regulatory T cells, antidepressants, sphingolipids, major depression

## Abstract

Genetic deficiency for acid sphingomyelinase or its pharmacological inhibition has been shown to increase Foxp3^+^ regulatory T-cell frequencies among CD4^+^ T cells in mice. We now investigated whether pharmacological targeting of the acid sphingomyelinase, which catalyzes the cleavage of sphingomyelin to ceramide and phosphorylcholine, also allows to manipulate relative CD4^+^ Foxp3^+^ regulatory T-cell frequencies in humans. Pharmacological acid sphingomyelinase inhibition with antidepressants like sertraline, but not those without an inhibitory effect on acid sphingomyelinase activity like citalopram, increased the frequency of Foxp3^+^ regulatory T cell among human CD4^+^ T cells *in vitro*. In an observational prospective clinical study with patients suffering from major depression, we observed that acid sphingomyelinase-inhibiting antidepressants induced a stronger relative increase in the frequency of CD4^+^ Foxp3^+^ regulatory T cells in peripheral blood than acid sphingomyelinase-non- or weakly inhibiting antidepressants. This was particularly true for CD45RA^−^ CD25^high^ effector CD4^+^ Foxp3^+^ regulatory T cells. Mechanistically, our data indicate that the positive effect of acid sphingomyelinase inhibition on CD4^+^ Foxp3^+^ regulatory T cells required CD28 co-stimulation, suggesting that enhanced CD28 co-stimulation was the driver of the observed increase in the frequency of Foxp3^+^ regulatory T cells among human CD4^+^ T cells. In summary, the widely induced pharmacological inhibition of acid sphingomyelinase activity in patients leads to an increase in Foxp3^+^ regulatory T-cell frequencies among CD4^+^ T cells in humans both *in vivo* and *in vitro*.

## Teaser

In their study, Wiese et al. found that pharmacological inhibition of acid sphingomyelinase with antidepressants such as sertraline or amitriptyline increased the proportion of Foxp3^+^ regulatory T cells among human CD4^+^ T cells both in cell culture *in vitro* as well as *in vivo* in patients treated for major depression.

## Graphical Abstract

**Graphical Abstract:**
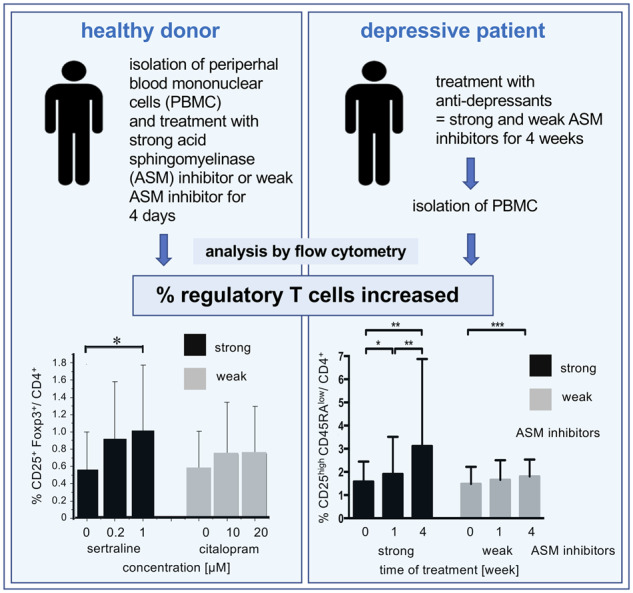

## Introduction

T cells are activated through cell-surface receptors such as the T-cell receptor and the co-stimulatory molecule CD28.^1^ In the cell membrane, these activating receptors are, of course, in close contact to surrounding phospholipids^2^ of which about 30% are sphingolipids.^3^ Sphingolipids are not chemically inert, but are further metabolized.^4^ The most prominent and complex sphingolipid sphingomyelin can be reversibly cleaved into ceramide and phosphocholine by sphingomyelinases including acid sphingomyelinase (ASM). In resting T cells, the Asm is localized at the inner leaflet of the lysosomal membrane where Zn^2+^ ions and acidic pH guarantee optimal enzymatic activity.^4^ Upon ligation of, e.g., CD28^5^ and CD95^6^ ASM activity in lysosomes is increased and lysosomes fuse with the cell membrane exposing the ASM on the cell surface.^4^

Recently, it has been shown that mice deficient for the enzyme acid sphingomyelinase (Asm; gene: *Smpd1*) have higher frequencies of Foxp3^+^ regulatory T cells (Treg) among CD4^+^ T cells than their wild-type counterparts.^7^^,^^8^ Moreover, injection of the tricyclic antidepressant amitriptyline, which induces Asm degradation, into healthy wild-type C57BL/6 mice, also led to an increase in Treg frequencies among CD4^+^ T cells.^7^ Therefore, induction of pharmacological Asm degradation is suitable for shifting the balance between Treg and CD4^+^ Foxp3^−^ conventional T cells (Tconv) towards Treg. We demonstrated in a measles virus infection model in mice that this shift in the Treg: Tconv balance strengthens Treg-mediated suppression of anti-viral immunity *in vivo*.^7^ Therapeutically, autoimmune diseases or overshooting immunity may be suitable indications for the use of Asm-degrading drugs as immunomodulators strengthening the Treg over the Tconv compartment.

For major depression, an inflammatory component has also been identified,^9^ suggesting that immunomodulation might contribute to the therapeutic efficacy of some antidepressants. In line with this, Treg, identified according to CD4 and high CD25 expression, have been reported to be reduced in patients suffering from the disease^10^ and to increase during anti-depressive therapy.^11^ Within the human Treg compartment, a sub-population of long-lived, but less functionally active resting Treg (rTreg) and short-lived activated effector Treg (efTreg) can be distinguished according to the expression of CD45RA, CD25, Foxp3 and CTLA-4.^12^^,^^13^ rTreg and efTreg, however, do not reflect different lineages, but efTreg develop from rTreg after sufficient activation.^12^ So far, efTreg and rTreg and their response to therapy have not been differentially analysed in major depression.

As a consequence of their continuous activation due to their auto-reactivity, CD28 co-stimulation and interleukin (IL)-2 receptor signaling,^14–20^ Treg express the checkpoint receptor CTLA-4. CTLA-4 molecules are stored intra-cellularly, but permanently shuttle between these stores and the cell membrane with only a very small fraction of the total pool of molecules expressed at the cell surface at any given timepoint.^21^ In Treg from Asm-deficient versus wild-type mice, we noted that CTLA-4 turnover was increased,^7^ suggesting that changes in CTLA-4 turnover by ASM-deficiency might have an impact on Treg frequencies among CD4^+^ T cells.

In this study, we now analysed whether functional ASM inhibitors have similar effects on the frequencies of human Treg among CD4^+^ T cells as previously observed in mice. For this, we investigated the effects of strongly and weakly/no ASM-inhibiting antidepressants^22^ on human Treg and Tconv populations from healthy donors *in vitro.* We validated our *in vitro* findings in a clinical study, analysing CD4^+^ T-cell sub-sets from patients suffering from major depression and treated with ASM-inhibiting and -non-inhibiting antidepressants.

## Materials and methods

### Study approval

The clinical study protocol was approved by the local Ethics Committee of the Faculty of Medicine at the University of Würzburg (vote no. 128/15). All participants gave written informed consent according to the Declaration of Helsinki prior to study inclusion. Table 1 summarizes the clinical and sociodemographic features of patients (*n* = 60).

**Table 1:** Clinical and sociodemographic features of patients (*n* = 60) and their association with strongly or weakly/no ASM-degrading antidepressants

Characteristics	Strong ASM inhibitors	Weak/no ASM inhibitors	*P*-value
	(*n* = 27)	(*n* = 33)	
Sex			n.s.
Female, *N* (%)	11 (40.7)	16 (48.5)	
Male, *N* (%)	15 (55.6)	13 (39.4)	
Not specified, *N* (%)	1 (3.7)	4 (12.1)	
Age (±SD)	47.08 (±12.96)	43.93 (±13.88)	n.s.
Marital status			n.s.
Married, *N* (%)	11 (40.7)	9 (27.2)	
Single, *N* (%)	6 (22.3)	10 (30.3)	
Separated, *N* (%)	8 (29.6)	5 (15.2)	
Partnership, *N* (%)	1 (3.7)	5 (15.2)	
Not specified, *N* (%)	1 (3.7)	4 (12.1)	
Employment status			n.s.
Employed, *N* (%)	16 (59.3)	16 (48.5)	
Unemployed, *N* (%)	8 (29.6)	10 (30.3)	
Retired, *N* (%)	2 (7.4)	3 (9.1)	
Not specified, *N* (%)	1 (3.7)	4 (12.1)	
Body mass index (kg/m^2^) MW (±SD)	30.76 (±5.91)	30.72 (±8.87)	n.s.
Smoker, *N* (%)	6 (23.08)	11 (33.33)	n.s.
Age at onset (±SD)	31.48 (±11.93)	30.79 (±13.68)	n.s.
Number of episodes (±SD)	8.78 (±13.89)	3.77 (±3.75)	n.s.
Number of hospitalizations (±SD)	1.80 (±3.04)	1.48 (±1.78)	n.s.
History of suicide attempts *N* (%)	9 (37.5)	10 (34.5)	n.s.
Length of illness (in years) (±SD)	16.18 (±12.73)	12.92 (±11.21)	n.s.
Family history depression, *N* (%)	13 (52.0)	17 (58.6)	n.s.
HAM-D admission (±SD)	27.68 (± 6.15)	27.41 (±7.37)	n.s.
Response Week 4, *N* (%)	11 (40.0)	10 (30.3)	n.s.
HAM-D Week 4 (±SD)	14.84 (±5.80)	14.97 (±8.14)	n.s.

n.s. = not specified.

Inhibitors used were divided into the two categories according to their capacity to functionally inhibit ASM^22^: Strong inhibitors were amitriptyline, doxepin, clomipramine, maprotiline and sertraline. Weak inhibitors were citalopram/escitalopram, venlafaxine, bupropion, mirtazapine, amlodipin, trozodon, duloxetine and agomelatine. Five patients exclusively received electroconvulsive therapy.

### Clinical samples

We initially recruited a total of 70 depressed in-patients (mean age = 45.64 ± 13.43 SD, 45.8% female) within the first 2–5 days after admission to the Department of Psychiatry, Psychosomatics and Psychotherapy of the University Hospital, Würzburg. Published studies looking into the abundance of Treg in patients with major depression^10^^,^^11^ showed that 27 patients per group provided enough statistical power. The patients received treatment with antidepressants according to doctor’s choice within a clinical routine setting. During the study, 10 patients either dropped out or the blood specimens were of insufficient quality on at least one of the analysis time points. In our study, we, thus, analysed 27 patients who received strongly ASM-inhibiting and 33 who received weakly/no ASM-inhibiting antidepressants. The severity of depressive symptoms was assessed at admission and then weekly by trained raters using the 21-item Hamilton Depression Rating Scale.^23^ The inclusion criteria were >18 years and an at least moderate depressive episode (Hamilton Depression Rating Scale, ≥ 14) which was the cause for hospital admission and the focus of therapeutic interventions. Response to antidepressant treatment was defined as a  ≥ 50% reduction of the Hamilton Depression Rating Scale at Week 4.^24^ Exclusion criteria comprised depression due to drug or substance abuse, a non-controlled addiction, schizophrenia or schizoaffective disorder, systemic glucocorticoid treatment, severe general or neurological medical conditions or acute infections, monitored by differential blood count, CRP, renal function, liver enzymes and coagulation tests. A further reason for not including patients in the study were, apart from the already mentioned in- and exclusion criteria, lacking consent, which was mostly due to patients feeling that everything was too much or due to fear of having blood taken. Vital signs such as heart rate and blood pressure were monitored. Blood for the assessment of the CD4^+^ T cells frequencies was collected at 6 pm using ammonium-heparin (NH_4_-heparin) tubes (Sarstedt).

### Culture of human peripheral blood mononuclear cells: high density pre-culture and 4-day culture in the presence of acid sphingomyelinase inhibitors

PBMC were isolated and cultured at high density (1 × 10^7^ cells/ml in 1.5 ml/well of a 24-well flat-bottom-plate; Greiner) as described earlier.^25^ This high-density pre-culture was used to restore tonic TCR signalling, thus simulating tissue-like conditions and ensuring susceptibility to the action of immunomodulating agents.^25^

After this pre-culture, cells were further cultured at a concentration of 1 × 10^6^ cells/ml for 4 days in the presence of titrated concentrations of the ASM inhibitors sertraline or citalopram—either alone or together with anti-CD28-Fab (clone CD28.3; 1, 0.5, 0.25 µg/ml)^26^ or 1 µg/ml of the CTLA-4 inhibitor tremelimumab (Creative Biolabs). In the Treg-depletion experiment, as well as in the experiment with the addition of external C6-ceramide, an anti-CD3-antibody (LEAF purified anti-human CD3, Clone HIT3a, 0.1 µg/ml, Biolegend) was added to these cultures.

### CTLA-4 surface expression analysis by capture assay

Total isolated PBMC (2 × 10^5^ cells, 96-well-round bottom plate; Greiner) were cultured in the presence of anti-CD3 monoclonal antibody (mAb, LEAF purified anti-human CD3, Clone HIT3a, 1 µg/ml, Biolegend), human IL-2 (Proleukin, Novartis, 0.1 µM) and sertraline (1 µM). To label (capture) the cell-surface-exposed CTLA-4, we added anti-CTLA-4 PE or a matching PE isotype control (both Biolegend) to the culture medium and incubated the cells at 37°C for 24 h. Afterwards, the cells were stained for the expression of CD4, CD25, Foxp3 and CD45RA. To determine the total CTLA-4 amount per cell, parallel samples were first fixed and permeabilized before staining for the expression of CTLA-4.

### Quantification of CTLA-4-mediated transendocytosis

Mouse embryonic fibroblasts expressing CD80 or CD80-mScarlet were seeded at a concentration of 1 × 10^4^ cells/well in a 48-well tissue culture plate (Greiner). Alternatively, mouse splenocytes were first activated with 1 µg/ml lipopolysaccharide (Sigma-Aldrich) overnight with a concentration of 1 × 10^7^ cells/ml in a 24-well plate. On the next day, after washing the cells, 1 × 10^6^ freshly isolated PBMCs were added and the cells were cultured for another 12 or 16 h, respectively, in the presence of 1 µM of sertraline or 1 µg/ml of the CTLA-4 inhibitor tremelimumab. An anti-CD3-antibody mAb (LEAF purified anti-human CD3, Clone HIT3a, 0.1 µg/ml, Biolegend) was also added to the cultures.

### Antibodies and flow cytometry

For fluorescence-activated cell sorting (FACS) analysis, cells were labelled with saturating amounts of antibodies in FACS buffer (PBS containing 0.1% BSA and 0.02% NaN_3_) to stain cell-surface antigens for 15 min on ice, followed by a fixation/permeabilization step (Fix/Perm buffer, eBioscience) for 30 min at room temperature and intracellular staining for 45 min at room temperature in permeabilization buffer (eBioscience). Stained cells were analysed on an LSR II flow cytometer (BD Biosciences).

For the staining of PBMC, the following Abs were used: Anti-CD3-PE-Cy7, anti-CD4-PerCP, anti-CD4-Pacific Blue, anti-CD4-FITC, anti-CD4-Alexa Fluor 700, anti-CD8-PE, anti-CD25-APC, mIgG_1_-APC, anti-CD45RA-PE, anti-CD45RA-PerCP-Cy5.5, anti-CD127-PE, anti-Foxp3-APC anti-Foxp3-Pacific Blue, anti-CCR7-Alexa Fluor 488, anti-Ki-67-Alexa Fluor 700, anti-CTLA-4-PE, anti-CTLA-4-PE-Cy7 (all Biolegend), anti-CD25-PE, anti-CD8-FITC, PE-Cy5-streptavidin, anti-CD86-biotin (all BD Bioscience) and viability dye (Thermo Fischer).

For di-4-ANEPPDHQ (ANE) staining, PBMC were incubated with 4 mM of ANE (Invitrogen) together with anti-CD4 APC-Cy7, anti-CD45RA BV510 and anti-CD25 APC (all from Biolegend) in RPMI medium for 30 min at 37°C and were immediately analysed by FACS.

### Quantification of C6-ceramide incorporation into human CD4^+^ T-cell sub-sets

For the click staining, 5 × 10^6^ PBMC were washed once with HBSS (Hanks Solution, Sigma Aldrich). Pellets were re-suspended in 100 µl of HBSS containing 25 µM ω-N_3_-C6-ceramide (6-azido-*N-*(*(2S, 3 R, E)*-1,3-dihydroxyoctadec-4-en-2-yl)hexanamide)^27^ and then further processed as described earlier.^28^^,^^29^ After the final three washing steps with HBSS, cells were immediately analysed by FACS.

### Isolation of CD4^+^ T cells and CD4^+^ CD25^+^ regulatory T cells from human PBMC and ASM activity assay

For the isolation of CD4^+^ cells, the CD4^+^ T-Cell Isolation Kit human from Miltenyi Biotec was used according to the manufacturer’s instructions and the purity was analysed by flow cytometry. For the isolation of CD4^+^ CD25^+^ (Foxp3^+^) Treg cells, the CD4^+^ CD25^+^ CD127^dim/^^−^ Regulatory T-Cell Isolation Kit II human from Miltenyi Biotec was used to separate Treg and Tconv from pre-enriched CD4^+^ T cells. The purity of the Treg was >95% (CD25^+^ Foxp3^+^) as analysed by flow cytometry. In brief, 3 × 10^5^ Treg or Tconv per condition were then cultured for 2 h at 37°C/5% CO_2_ in the absence or presence of sertraline at 0.2, 1, 5 and 10 µM. After the short-term culture, the T cells were disrupted by repeated freezing and thawing and further processed as described earlier.^7^

### Depletion of regulatory T cells from total PBMC

CD4^+^ CD45RA^+^ CD25^+^ resting and CD4^+^ CD45RA^−^ CD25^high^ effector Treg cells were depleted from total PBMC using the BD FACS Aria III. The success of the depletion process was confirmed by flow cytometric analysis (>90% purity for CD4^+^ and >95% purity for Treg-depleted cells). For stimulation of the cells after depletion, anti-CD3 mAb HIT3a was added to the cell culture (concentration, 0.1 µg/ml; Biolegend).

### Statistical analysis

For parametric data, either a two-tailed unpaired Student’s *t*-test (two groups) or a one- or two-way ANOVA followed by Tukey’s post-test (more than two groups) was performed (OriginPro 2016 G, OriginLab and Prism 7, GraphPad Software). Differences were considered to be significant when *P* < 0.05. All summary data are displayed as means and SD unless indicated otherwise.

General linear models, with and without repeated measures, were applied to analyse associations of CD4^+^ T cell frequencies, the type of antidepressants (strong versus weak/no ASM inhibition) and response after 4 weeks of treatment with the antidepressants. Clinical and sociodemographic variables were compared between patients with strong versus weak/no ASM activity-inhibiting antidepressants with *t*-tests for independent samples in case of quantitative data and with Fisher exact or Pearson’s chi-square tests in case of qualitative data. The analyses were performed using SPSS for Windows (Releases 25, SPSS Inc.). Only complete data sets were included in the analysis.

### Data availability

The data presented in this study are available from the corresponding author upon request.

## Results

### Sertraline treatment increases Treg frequencies among human CD4^+^ T cells *in vitro*

**Figure 1:**
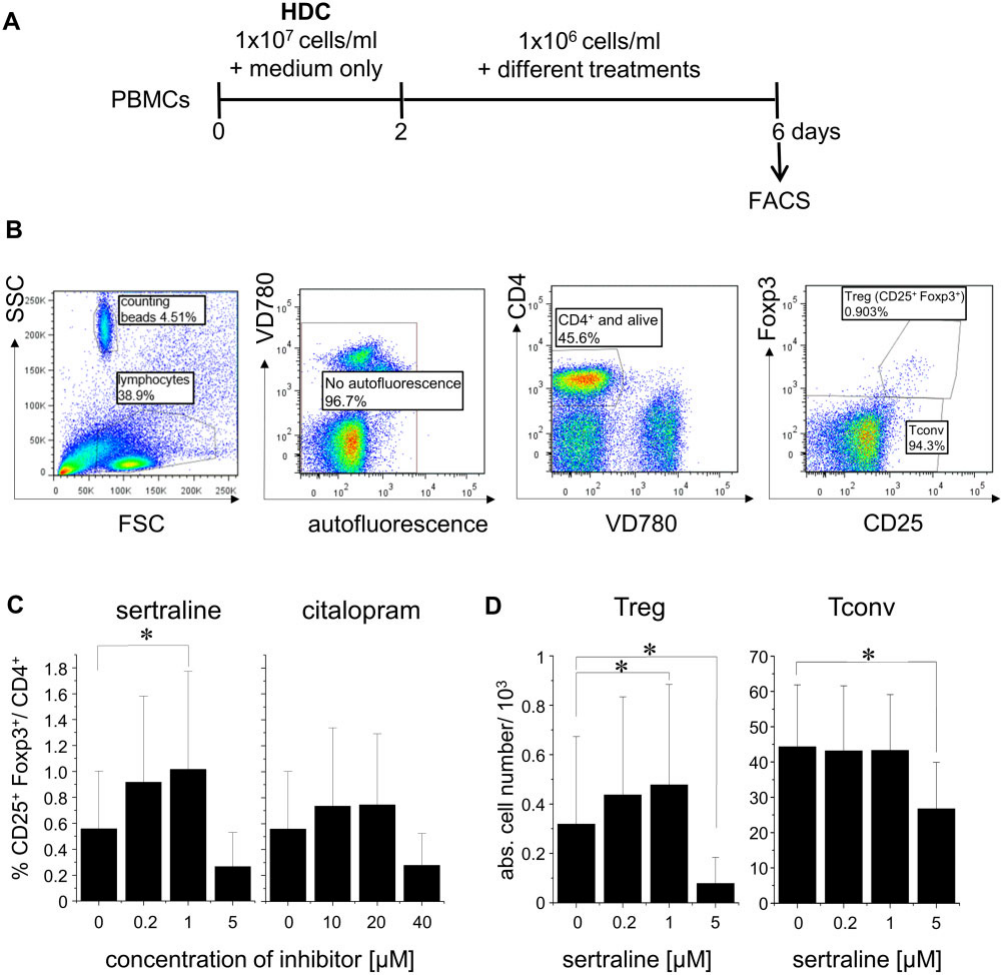
**Functional ASM inhibition in human PBMC *in vitro* increases Treg frequencies among CD4^+^ T cells.** (**A**) Schematic representation of the experimental set-up. (**B**) Gating strategy used for FACS-analysis of human PBMC after 6 days of culture, i.e. 2 days at high (10^7^ cells/ml) and 4 days at normal cell concentrations (1 × 10^6^ cells/ml). FSC/SSC: linear scale; fluorescence: log_10_ scale. (**C**) Frequencies of CD25^+^ Foxp3^+^ Treg among CD4^+^ T cells (*n* = 27; PBMC from independent healthy donors), and (**D**) absolute cell numbers of CD25^+^ Foxp3^+^ Treg (left) and CD25^−^ Foxp3^−^ Tconv (right) in the presence of increasing concentrations of sertraline or citalopram. *P < 0.05.

**Figure 2:**
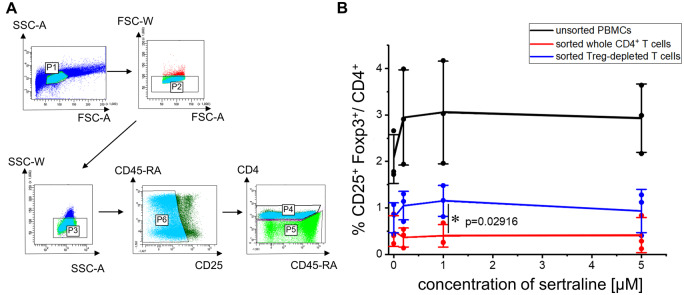
**ASM inhibition with sertraline increases the proportion of pre-existing Treg among CD4^+^ T cells.** (**A**) Gating strategy used for FACS-sorting of Treg-depleted CD4^+^ T cells (FSC/SSC: linear scale; fluorescence: log_10_ scale.). During the 4-day culture at normal cell density, we added 0.1 µg/ml anti-CD3 mAb to the PBMC as well as the ASM inhibitor sertraline. (B) Treg frequencies/CD4^+^ of unsorted PBMC, sorted total CD4^+^ T cells and Treg-depleted CD4^+^ T (*n* = 3 experiments). Individual values as well as means and standard deviations are shown. *P < 0.05.

To test whether functional ASM inhibition would increase the frequencies of Treg among human CD4^+^ T cells, we used human PBMC containing T cells with tissue-like responsiveness which is required for valid *in vitro* testing of immunomodulatory reagents (Fig. 1A).^25^ Among living CD4^+^ T cells (Fig. 1B), the frequencies of CD25^+^ Foxp3^+^ Treg increased from an average of 0.56% without inhibitor to 1.02% in the presence of 1 µM sertraline—a selective serotonin re-uptake inhibitor (SSRI) which potently blocks ASM’s activity (Fig. 1C, *n* = 27). This means that the proportion of Treg among CD4^+^ was increased by 82%, which is about the factor seen in patients recovering from severe depression^11^ and, regarding other diseases, e.g. in patients with rheumatoid arthritis responding to tumournecrosis factor inhibitors.^30^ We observed similar relative increases in Treg frequencies when adding sertraline to PBMC stimulated with sub-optimal amounts of anti-CD3 mAb (Fig. 2, data not shown). The weakly ASM-inhibiting SSRI citalopram did not increase the proportion of Treg among CD4^+^ T cells (Fig. 1C). This was in contrast to amitriptyline, a tricyclic antidepressant with strong ASM-inhibitory activity for which we also saw an increase in the frequency of Treg among CD4^+^ T cells *in vitro.* The optimal concentration of amitriptyline for an increase in the proportion of Treg/CD4^+^ varied for cells from different donors, which was not the case for sertraline (Supplementary Fig. 1 and Supplementary Methods) (Fig. 1C).

The increase in Treg frequencies among human CD4^+^ T cells induced by 0.2 and 1 µM of the ASM inhibitor sertraline was due to higher absolute numbers of Treg (Fig. 1D), whereas Tconv numbers were not affected at these concentrations of the SSRIs. High concentrations of the inhibitors, 5 µM of sertraline (Fig. 1D) and 40 µM of citalopram (data not shown), were clearly toxic for Treg and Tconv, decreasing their absolute numbers.

As Foxp3 and CD25 are also expressed to some degree by non-regulatory memory CD4^+^ T cells,^12^ we compared Treg-depleted (sorted) and -sufficient human PBMC *in vitro*. As a control for sort-related stress, we included PBMC stained and run through the cell sorter without, however, separating sub-populations. Only the populations containing pre-existing Treg showed an increase in Treg frequencies among CD4^+^ T cells after ASM inhibition (Fig. 2). Therefore, functional inhibition of the ASM in human PBMC did not convert Tconv into Foxp3^+^ Treg, but led to an increase in the number of pre-existing Treg among CD4^+^ T cells *in vitro*.

### Treatment of patients with ASM-inhibiting antidepressants increased Treg frequencies among CD4^+^ T cells *in vivo*

**Figure 3:**
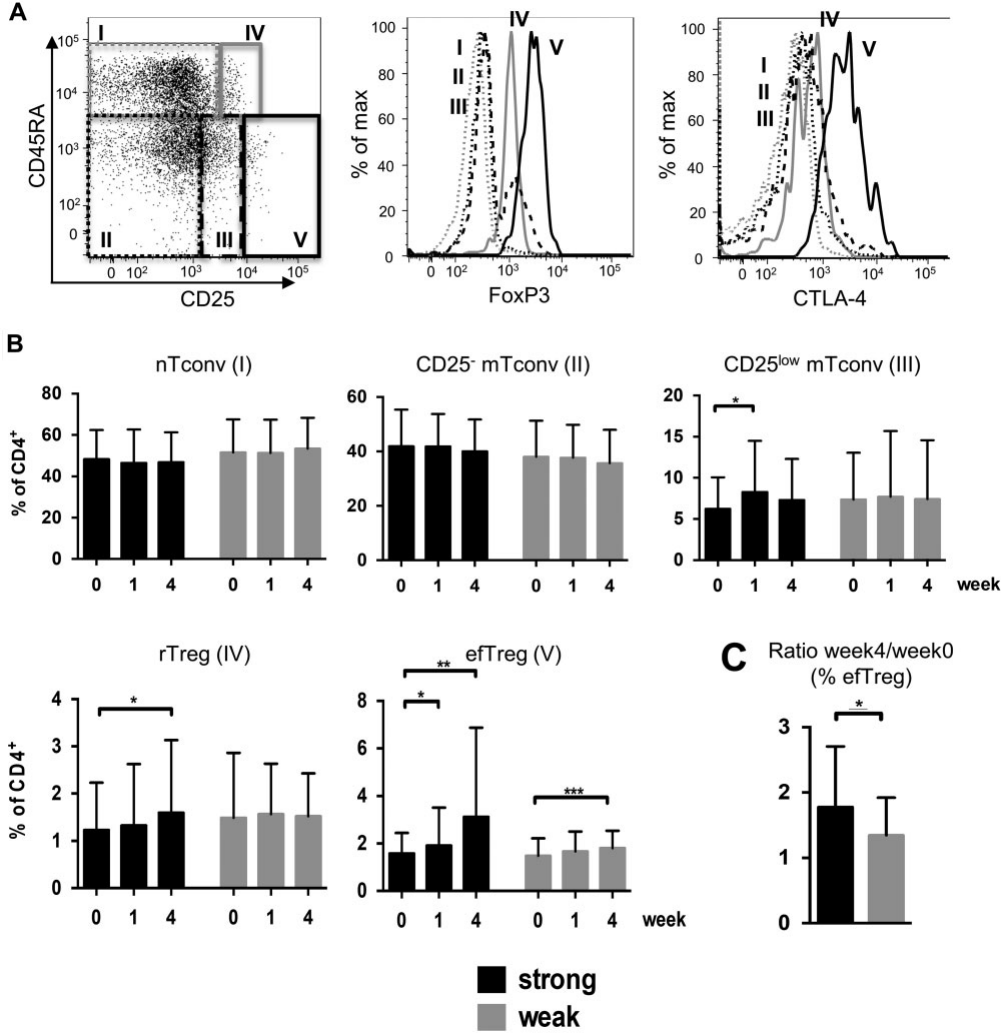
**Increase in Treg frequencies among CD4^+^ T cells in patients treated for major depression with ASM inhibitors.** (**A**) Gating of CD4^+^ T-cell sub-sets using CD45RA and CD25 to identify nTconv (I), CD25^−^ mTconv (II), CD25^low^ mTconv (III), rTreg (IV) and efTreg (V) among CD4^+^ T cells of patients with major depression (dot plot, log_10_ scale). Expression of Foxp3 and CTLA-4 was used to control for correct sub-set gating (histograms). (**B**) Summary graphs of the frequencies of the indicated CD4^+^ T-cell sub-populations over time in patients receiving strongly ASM-inhibiting antidepressants (black; *n* = 27) or weakly/no ASM-inhibiting antidepressants (grey; *n* = 33). A repeated measures ANOVA followed by a Tukey’s *post-hoc* test revealed significant changes among CD25^low^ mTonv, rTreg and efTreg during the course of treatment. In addition, a repeated measures ANOVA showed a significant contrast (*F* = 10.566; d*f* = 2; *P* < 0.001) during the course of 4 weeks with a higher increase in efTreg frequencies/CD4^+^ in patients receiving strong versus patients receiving weakly/no ASM-inhibiting antidepressants. (**C**) Ratio of efTreg frequencies/CD4^+^ at Week 4 over Day 0 for patients receiving strongly (black; *n* = 27) or weakly/no (grey; *n* = 33) ASM-inhibiting antidepressants. *P < 0.05, **P < 0.01, ***P < 0.001.

Knowing that ASM inhibition caused an increase in Treg frequencies among mouse CD4^+^ T cells *in vitro* and *in vivo*, and among human CD4^+^ T cells *in vitro* (Figs. 1 and 2), we prospectively analysed Treg frequencies among CD4^+^ T cells in blood samples from 60 patients undergoing treatment for major depression. It is worth noting that there were no significant clinical or sociodemographic differences between the patients treated with strong versus weak/no ASM-inhibiting antidepressants (Table 1). Directly *ex vivo*, human Treg are either of a resting (r, CD45RA^+^ CD25^low^) or an effector (ef, CD45RA^−^ CD25^high^) phenotype.^12^ We therefore quantified T-cell sub-populations by flow cytometry according to the expression of CD45RA and CD25 (Fig. 3A; sub-populations I–V). To confirm correct gating of sub-populations, we determined the expression of Foxp3 and CTLA-4 (Fig. 3A, histograms). We then stratified patients according to the anti-depressive regimen they had received, distinguishing between patients on strongly versus weakly/no ASM-inhibiting drugs.^22^ Of the 60 patients, 27 received strongly (Fig. 3B, black columns) and 33 weakly/no ASM-inhibiting antidepressants (Fig. 3B, grey columns). We observed an increase in the proportion of rTreg and efTreg among CD4^+^ T cells after 4 weeks of treatment with strong ASM-inhibiting antidepressant compared to baseline (Fig. 3B, black columns, gates IV and V). Although rTreg frequencies were not increased among CD4^+^ T cells of patients receiving weakly/no ASM-inhibiting antidepressants (Fig. 3B, grey columns, gate IV), the proportion of efTreg was also higher in this cohort after 4 weeks of treatment (Fig. 3B, grey columns, gate V). When comparing the increase in %efTreg/CD4^+^ T cells in both patient cohorts, patients receiving strong ASM-inhibiting antidepressants displayed a significantly higher increase (mean, 78%) than patients receiving weakly/no ASM-inhibiting antidepressants (mean, 35%; Fig. 3C). Differences were significant due to the paired nature of the statistical testing. Analysis of changes in Treg frequencies of individual patients corroborated the conclusions (Supplementary Fig. 2A and B). Among Tconv, we noted a transient increase in CD25^low^ memory cells after 1 week of strong functional ASM inhibitor treatment, whereas nTconv and CD25^−^ mTconv frequencies were not altered (Fig. 3B). Hence, our clinical data indicate that Treg frequencies among CD4^+^ T cells *in vivo* increased particularly well in patients receiving drugs strongly inhibiting ASM activity.

As depressed mood in patients with major depression is associated with low Treg frequencies among CD4^+^ T cells,^11^ we also assessed whether the more pronounced increase in Treg frequencies among CD4^+^ T cells upon treatment with strongly versus weakly/no ASM-inhibiting antidepressants might translate into better clinical improvement. Comparing both groups of patients using the Hamilton Depression Rating Scale, we, however, observed no differences in the clinical response between patients receiving strongly versus weakly/no ASM-inhibiting antidepressants (Supplementary Fig. 2 C).

### Susceptibility to ASM inhibitors and C6-ceramide distinguishes Treg from Tconv

**Figure 4:**
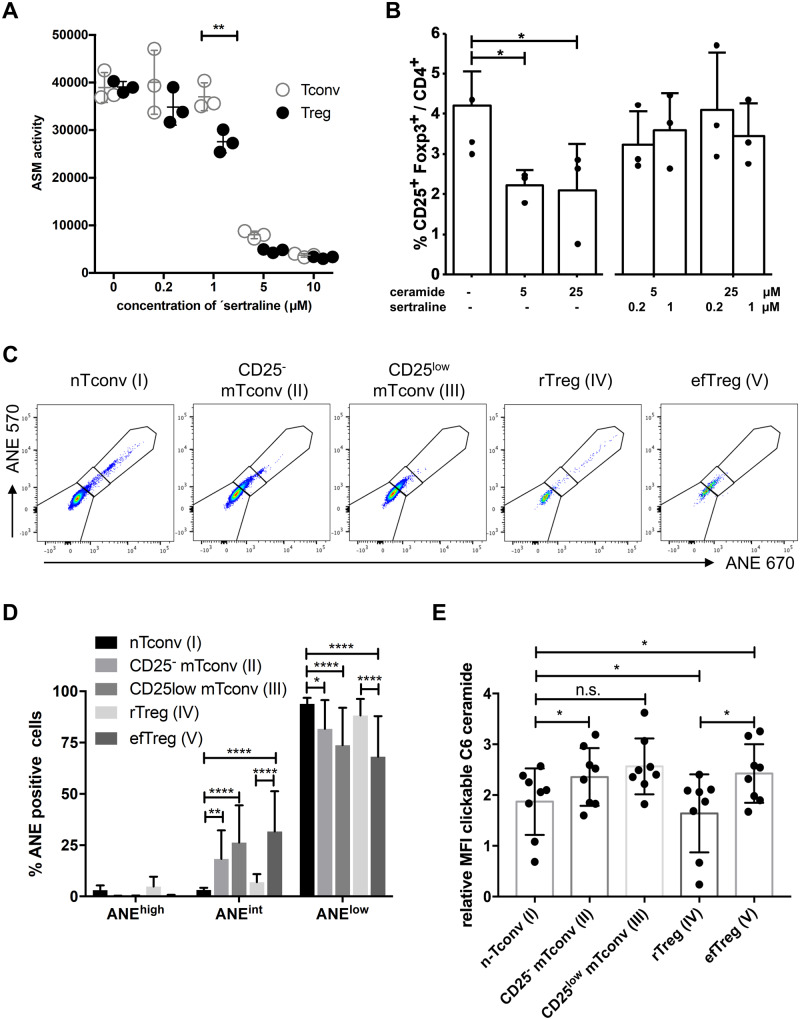
**ASM activity, response to C6-ceramide and membrane lipid order of different human Treg and Tconv sub-sets.** (**A**) ASM enzyme activity in enriched human CD4^+^ Treg and Tconv cells after addition of ASM inhibitors. *N* = 3 technical replicates together with means and standard deviations are shown. Two-way ANOVA (*P* < 0.01) followed by Sidak’s multiple comparisons test. The experiment was repeated with similar result. (**B**) Frequencies of Treg among CD4^+^ T cells after culture in the presence of anti-CD3 mAb (0.1 µg/ml) and 0, 5 or 25 µM C6-ceramide, 0, 0.2 or 1 µM sertraline and 0, 1 or 10 µM citalopram. *N* = 3 individual experiments. Columns represent means + SD. (**C**) Representative data showing detection of the ANE dye at 570 versus 670 nm after incorporation into the different T-cell sub-sets by FACS (log_10_ scale). (**D**) Summary of ANE dye incorporation (*n* = 15). (**E**) Chemical structure of azido-functionalized C6-ceramide and graph summarizing incorporation of the azido-functionalized C6-ceramide into the indicated CD4^+^ T-cell sub-sets. Click chemistry was used to detect the fed ceramide in the cell membrane. *n* = 8 independent experiments. Individual values as well as means and standard deviations are shown. *P < 0.05, **P < 0.01, ***P < 0.001.

In order to define the mechanism responsible for the increase in Treg frequencies/CD4^+^ due to ASM inhibition, we first analysed ASM activities in human Treg and Tconv. In sorted CD4^+^ CD25^high^ Treg (>95% CD25^+^ Foxp3^+^), basal ASM activity was similar to sorted Tconv (Fig. 4A). ASM activity in Treg was, however, more susceptible to sertraline-mediated inhibition compared to Tconv (Fig. 4A). As expected,^31^ treatment of human CD4^+^ T cells with sertraline or amitriptyline reduced ASM protein amounts due to lysosomal degradation (Supplementary Fig. 5 and Supplementary Methods).

We hypothesized that ASM inhibition induces a relative increase in the number of Treg due to lowering ASM-catalyzed ceramide production. To functionally test this hypothesis, we added C6-ceramide to our cultures and again monitored Treg and Tconv (Fig. 4B). In contrast to ASM inhibition, addition of C6-ceramide decreased Treg frequencies among CD4^+^ T cells (Fig. 4B). This decrease was due to a stronger reduction in Treg numbers (74% in the presence of 5 µM C6-ceramide versus control) than in Tconv (49% in the presence of 5 µM C6-ceramide versus control). Together, these data show that Treg respond more strongly than Tconv to increased concentrations of ceramide in cellular membranes.

Having established that ceramide concentrations in cellular membranes are a key rheostat of the Treg/Tconv balance in human T cells, we studied sphingolipid composition. Therefore, we prepared total membranes from both FACS-sorted Treg and Tconv using similar gates as for Fig. 3 (Supplementary Fig. 3A) and quantified different ceramide and also sphingomyelin species by mass spectrometry. In contrast to mouse Treg,^7^^,^^32^ we observed that human efTreg contained less total ceramide than Tconv which was mainly due to reduced amounts of C16 and C24:1 ceramide (Supplementary Fig. 3B and Supplementary Methods). This suggests that ASM activity in human Treg might be more strongly counteracted by higher activity of cellular ceramidases or less *de novo* ceramide production in human Treg compared to Tconv.

### Lower lipid membrane order characterize both naïve/resting versus effector/memory Treg and Tconv

In mice, increased Asm activity in Treg versus Tconv was associated with less cells of high lipid order in their cell membranes among Treg compared to Tconv.^7^ We measured membrane lipid order using the ANE dye detected at 670 and 570 nm (Fig. 4C and D, dot plots and summary bar graph, respectively). In comparison to nTconv (I) and rTreg (IV), efTreg (V) contained less cells of high lipid order excluding the ANE dye (ANE^low^) and more cells of intermediate lipid order. Similarly, CD25^−^ mTconv (II) and CD25^low^ mTconv (III) also contained less cells of high lipid order than nTconv, which is also reminiscent of our findings in mice.^7^ Independent of the T-cell lineage, fewer cells with high lipid order, thus, correlated with effector/memory differentiation as efTreg also harboured less cells with high lipid order than rTreg. Moreover, reduced membrane lipid order in mouse T cells was reflected by a higher capacity to incorporate ceramide into the cell membrane.^28^^,^^29^ The same was true for human CD4^+^ T cells with effector/memory Treg or Tconv incorporating more ceramide than resting/naïve Treg or Tconv (Fig. 4E). Therefore, effector/memory differentiation positively correlated with an increased proportion of cells with low membrane lipid order among human Treg and Tconv.

### Acid sphingomyelinase inhibition increases CTLA-4 turnover in Treg, but not transendocytosis of ligand CD80 and CD86

**Figure 5:**
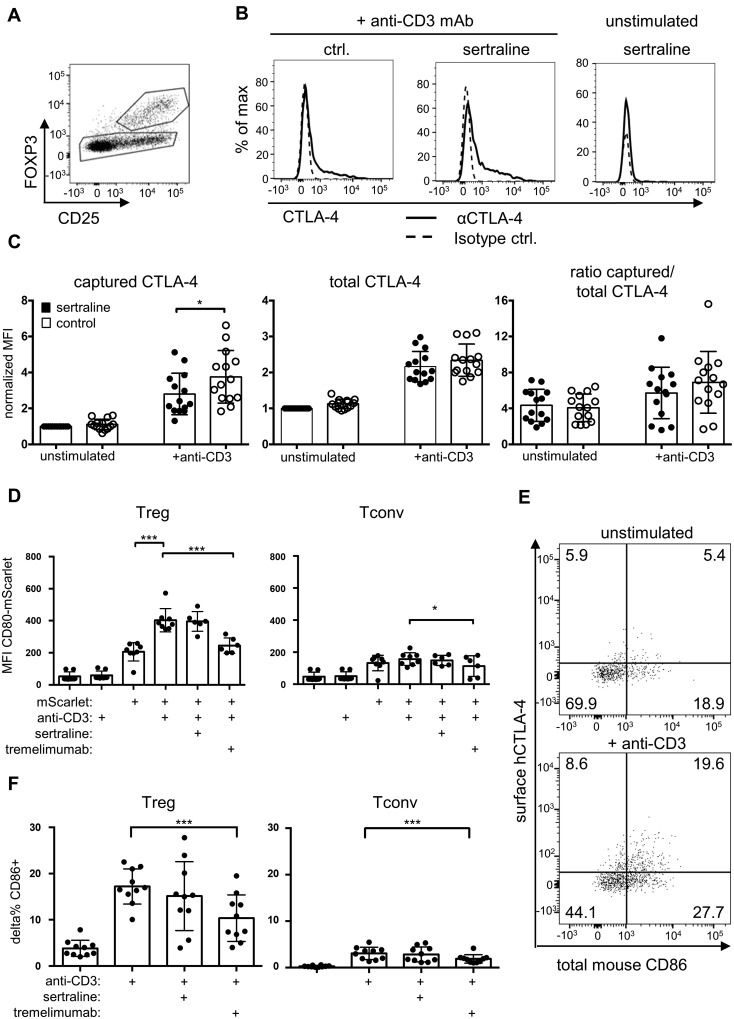
**Functional ASM inhibition by sertraline modulates CTLA-4 turnover but not CTLA-4-mediated transendocytosis of ligands.** (**A**) Gating strategy to detect Treg among human PBMC cultured for 24 h in the presence or absence of sertraline as well as an anti-CD3 mAb (log_10_ scale). (**B**) An anti-CTLA-4 mAb was added to ‘capture’ CTLA-4 molecules appearing on the cell surface (log_10_ scale). (**C**) Summary graph of anti-CTLA-4 mAb acquired by Treg via the cell surface (left), total CTLA-4 expression by Treg (middle) and ratio of anti-CTLA-4 mAb captured on the cell surface/24 h to total CTLA-4 expression (right) (*n* = 14–17). (**D**) Transendocytosis of mouse-CD80-mScarlet molecules expressed by mouse embryonic fibroblasts to human Treg (left) or Tconv (right). Ligand binding by CTLA-4 was blocked with anti-CTLA-4 mAb tremelimumab (*n* = 4–6). (**E**) Representative dot plots of gated human CD4^+^ mouse B220^−^ human Foxp3^+^ Treg cells. The dot plots show cell-surface expression of human CTLA-4 versus total (surface and intra-cellular) mouse CD86 (log_10_ scale). Numbers indicate percent cells/quadrant. (**F**) Transendocytosis of mouse CD86 from mouse splenic lipopolysaccharide blasts to human Treg (left) or Tconv (right) (*n* = 10). (C, D, F) Individual values as well as means and standard deviations are shown. **P < 0.01, ***P < 0.001.

Studying mouse Treg, we had observed that Treg from Asm-deficient mice had a higher turnover of the Treg effector molecule CTLA-4^33–35^ in the cell membrane than Treg from wild-type mice.^7^ Similarly, in the presence of sertraline, more CTLA-4 molecules were transported to the surface of human Treg within 24 h of CD3 stimulation (Fig. 5A–C, left panel). This increase in CTLA-4 molecules exposed at the surface of sertraline-treated Treg compared to controls appeared to be mainly due to a higher fraction of the total pool of CTLA-4 molecules appearing at the cell surface (Fig. 5C, right panel) rather than increased overall expression (Fig. 5C, middle panel).

Once on the cell surface, CTLA-4 binds CD80 and CD86 on antigen-presenting cells, removes these molecules from their surface and internalizes them—a process called transendocytosis—which is crucial for the inhibitory activity of CTLA-4.^21^^,^^36^^,^^37^ Functional ASM inhibition by sertraline, however, did not modulate CTLA-4-mediated acquisition of CD80 molecules from mouse embryonic fibroblasts expressing mouse(m)CD80-mScarlet fusion protein compared to control-treated Treg (Fig. 5D). We repeated the transendocytosis assay using lipopolysaccharide-pre-activated mouse splenocytes as CD86 donors. Also here, sertraline did not modulate transendocytosis of CD86 from mouse splenocytes into human Treg (Fig. 5E and F). Thus, despite increasing CTLA-4 turnover, ASM inhibition did not alter CTLA-4-mediated transendocytosis of CD80 and CD86.

### ASM inhibition requires CD28 co-stimulation to increase Treg frequencies among human CD4^+^ T cells

**Figure 6:**
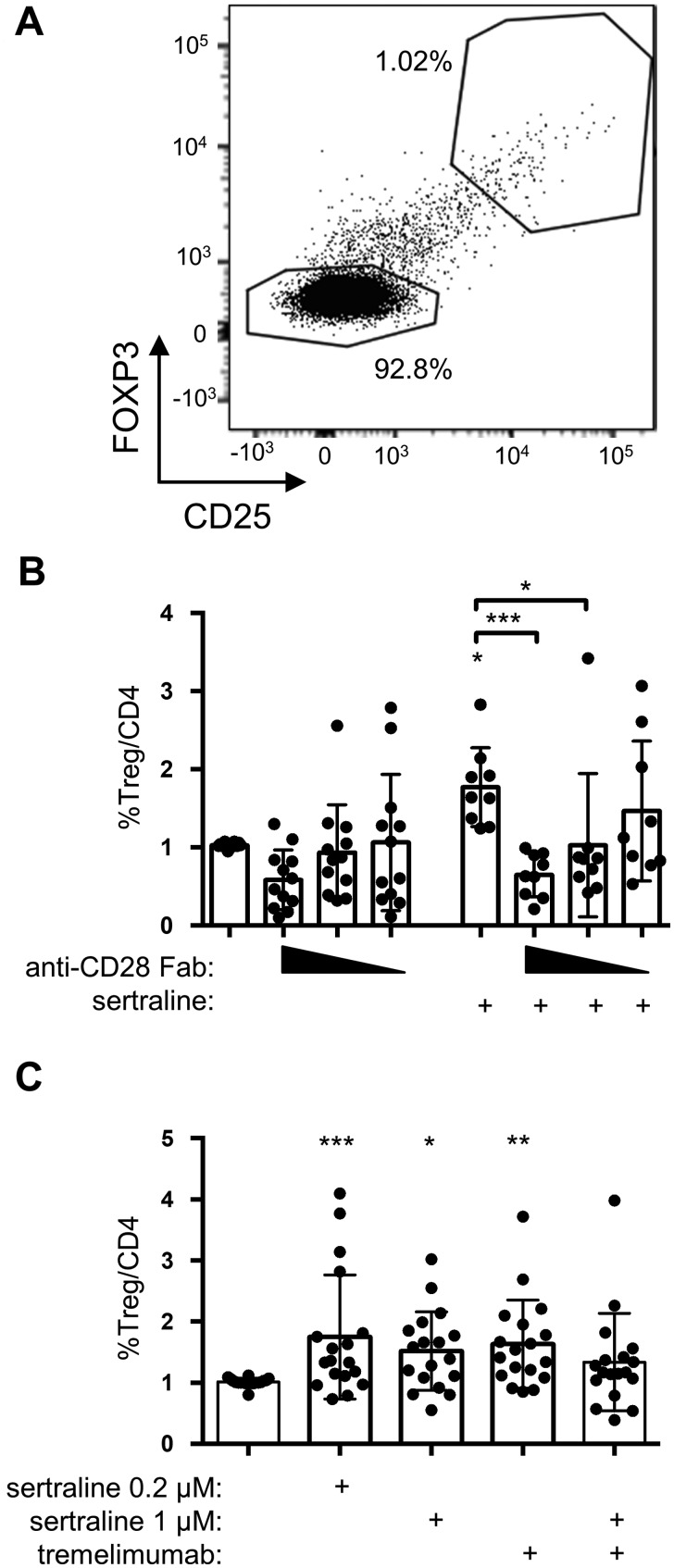
**ASM inhibition-induced increase in Treg frequencies/CD4^+^ T cells is CD28-dependent.** (**A**) Gating strategy to detect efTreg-like cells in our cultures (Fig. 2) defined as the top 1% regarding CD25 and Foxp3 expression (log_10_ scale). (**B**) CD28 co-stimulation was inhibited by using anti-CD28 Fab (1, 0.5 and 0.25 µg/ml). Sertraline was added at 1 µM (*n* = 7–12). (**C**) To block CTLA-4 function, anti-CTLA-4 mAb tremelimumab (1 µg/ml) was added to the cultures (*n* = 18). (B, C) Individual values as well as means and standard deviations are shown. *P < 0.05, **P < 0.01, ***P < 0.001.

CTLA-4 shares its ligands with the co-stimulatory molecule CD28 on T cells whose crosslinking with a monoclonal antibody has been shown to strongly induce ASM activity in T cells.^5^ As CD28 co-stimulation also crucially contributes to the balance of Treg and Tconv,^14^^,^^15^^,^^17^^,^^18^ we analysed whether it was required for the increase in the proportion of Treg/CD4^+^ T cells upon ASM inhibition. Adding an anti-CD28 Fab fragment inhibiting ligand binding to CD28^26^ to human PBMC prevented the increase in Treg frequencies among CD4^+^ T cells otherwise induced by inhibition of ASM (Fig. 6A and B). Analysis of the proliferation marker Ki-67, further, showed that co-stimulation of CD28 did not impact proliferation of Tconv and Treg under these culture conditions, in turn indicating that CD28 co-stimulation promoted survival of Treg in culture (Supplementary Fig. 4). To further test whether CD28 (co-) stimulation was the driver of the increase in Treg frequencies/CD4^+^ T cells upon ASM inhibition, we functionally blocked CTLA-4, a negative regulator of CD28 signalling and constitutively expressed at high levels by all efTreg (Fig. 3).^12^ To focus on efTreg, we adjusted our gating for each patient to the top 1% according to the expression of CD25 and Foxp3 in control cultures without sertraline. Inhibiting CTLA-4 with a blocking antibody in clinical use^37^ increased the proportion of Treg among CD4^+^ T cells *in vitro* (Fig. 6C). Culturing human PBMC in the presence of both the sertraline and the anti-CTLA-4 mAb did not have an additive effect (Fig. 6C). ASM inhibition, thus, increased Treg frequencies among human CD4^+^ T cells *in vitro* by enhancing the positive impact of co-stimulation of CD28 on the Treg compartment.

## Discussion

In this study, we analysed the impact of pharmacological ASM inhibition on the balance of human Treg and Tconv. We found that inhibition of ASM activity increases the frequencies of Treg among CD4^+^ T cells of healthy blood donors *in vitro* (Figs. 1 and 2) and in patients with major depression treated with ASM-inhibiting versus non-inhibiting antidepressants *in vivo* (Fig. 3). Mechanistically, we noted that (co-)stimulation of CD28 was required for ASM inhibition to increase Treg frequencies among human CD4^+^ T cells *in vitro* (Fig. 6). Our data, thus, provide evidence that the capacity to increase Treg frequencies among human CD4^+^ T cells *in vivo* is part of the mode of action of ASM-inhibiting drugs in routine clinical use.

Concerning the specificity of sertraline and other tricyclic antidepressants for the ASM one caveat is that they also functionally inhibit the acid ceramidase due to their amphiphilic properties in the acidic environment of the lysosome.^38^ However, adding C6-ceramide to human PBMC decreased Treg frequencies/CD4^+^ (Fig. 4B). This showed that human Treg not only ‘tolerate’ less ceramide than Tconv (Supplementary Fig. 3), but that they are also very sensitive to changes in ceramide content. Moreover, the ceramide pool controlled by the ASM clearly matters for Treg homeostasis as ASM inhibition fully compensated the effect of externally added C6-ceramide (Fig. 4B).

As part of the transcriptional program controlled by Foxp3, expression of sphingomyelin synthase 1 (SMS1 encoded by the *Sgms1* gene) is reduced in Treg, but not Tconv.^32^ SMS1 generates ceramide which sequesters the ‘inhibitor 2 of PP2A’ (‘I2PP2A’; also called ‘SET’) and, thus, activates the phosphatase PP2A. PP2A then inhibits the mTOR/AKT pathway in Treg.^32^ Also ceramide generated by (bacterial) sphingomyelinase reduces mTOR/AKT signalling in Treg.^39^ Therefore, ASM inhibition might enhance mTOR/AKT signalling which is a key pathway activated upon CD28 ligation.^1^ As Treg, in contrast to Tconv, strongly rely on CD28 signalling for their survival and function under steady-state conditions^14^^,^^15^^,^^17^^,^^18^ changes in mTOR/AKT signalling efficiency can be expected to preferentially impact the Treg versus the Tconv compartment. We, thus, think that ASM inhibition increases CD28 signalling in Treg through enhanced mTOR/AKT signal transduction.

In contrast to human CD4^+^ T cells, the increase in the proportion of Treg among mouse CD4^+^ T cells upon ASM inhibition *in vitro* required addition of IL-2 to the cell cultures^7^ (unpublished data). IL-2, thus, relatively protected mouse Treg as compared to Tconv from cell death induced upon ASM inhibition. The differential dependence on IL-2 (mouse) versus CD28 signalling (human) for Treg to preferentially survive upon ASM inhibition as compared to Tconv offers a key to understand the reason why this preferential survival was mainly due to less cell death (mouse) versus better survival of Treg (human) compared to Tconv.

In line with the work of others, we found that blocking the key negative regulator of CD28 co-stimulation, i.e. CTLA-4, increased the proportion of (ef)Treg among human CD4^+^ T cells *in vitro* (Fig. 6).^33^^,^^35^ In these culture assays, CTLA-4 and ASM inhibition had no additive or synergistic effect further supporting the notion that ASM inhibition provides for better CD28 co-stimulation of Treg. However, CTLA-4 might also function as an ‘internal’ ASM inhibitor, meaning that blockade of CTLA-4 with tremelimumab might (partially) counteract sertraline-induced ASM inhibition.

As ASM inhibition had no effect on transendocytosis of CD80 and CD86 by CTLA-4 (Fig. 5D–F), we speculate that the higher turnover of CTLA-4 we had observed (Fig. 5A–C) might have interfered with CTLA-4-mediated recruitment of phosphatases to the cell membrane.^40^ This would enhance, of course, both T-cell receptor- and CD28-induced signalling.

Our understanding of the role of the immune system in major depression has steadily grown.^41^ Anti-inflammatory treatment approaches delivered beneficial effects^42^^,^^43^ although not in all patients,^44^ hence the identification of patients with disturbed inflammatory pathways has been advocated.^45^ In our clinical trial, patients receiving antidepressants with strong ASM-inhibiting capacity showed a more pronounced increase in efTreg frequencies/CD4^+^ than patients on antidepressants with no effect on ASM activity (Fig. 3). Moreover, the percentage of rTreg/CD4^+^ was increased only in patients on ASM-inhibiting antidepressants. This indicates that inhibiting ASM activity in humans increases Treg frequencies/CD4^+^*in vivo.* Patients receiving antidepressants with a weak/no effect on ASM activity, interestingly, also harboured higher frequencies of efTreg/CD4^+^ after 4 weeks of treatment compared to baseline (Fig. 3B). This may either be due to increases in serotonin levels induced by SSRIs or the result of multiple factors modulated due to clinical improvement of patients. So far it is unclear, which factors contribute to the low Treg frequencies/CD4^+^ in patients with major depression prior to treatment. For serotonin, however, a positive effect on human Treg *in vitro*^46^ has been shown, which may, of course, contribute to the increase in %Treg/CD4^+^ in patients on SSRIs.

Clinically, there were no differences in the response to therapy of patients receiving strongly versus weakly/no ASM-inhibiting antidepressants (Supplementary Fig. 2C). The reason for this might be that inflammation contributes to the pathogensis of major depression, but that alterations in neurotransmitter pathways are the main drivers of this disease. In addition and as already mentioned, efTreg frequencies/CD4^+^ also normalize in patients on non-ASM-inhibiting antidepressants, albeit later and excluding the rTreg compartment (Fig. 3B).

## Conclusion

Generally, our observations support the previous findings on the important role of inhibiting the ASM in the treatment response to antidepressants.^47^ As far as the clinical use of ASM-inhibiting drugs is concerned, our data indicate that the positive effects of antidepressants on the Treg compartment in humans may be further exploited for the treatment of *bona fide* autoimmune and inflammatory diseases. For example, in multiple sclerosis and rheumatoid arthritis, defects in Treg numbers and function have been described,^30^^,^^48^ indicating that patients could possibly benefit from taking Treg-boosting drugs. In line with this projection, a recent article^49^ has shown that ASM-deficient mice are protected from experimental autoimmune encephalomyelitis compared to wild-type controls probably due to the increased Treg activity in the Asm-deficient animals.^7^ Furthermore, a double-blind placebo-controlled clinical trial in patients with multiple sclerosis found the ASM inhibitor fluoxetine significantly reduced the number of active inflammatory brain lesions on magnet resonance imaging.^50^

## Supplementary material

Supplementary material is available at *Brain Communications* online.

## Supplementary Material

fcab020_Supplementary_Data

## Abbreviations

ASM/Asm =: acid sphingomyelinase (human/mouse)
efTreg =: effector CD4^+^ Foxp3^+^ regulatory T cell
FACS =: fluorescence-activated cell sorting
IL =: interleukin
mAb =: monoclonal antibody
PBMC =: peripheral blood mononuclear cells
rTreg =: resting CD4^+^ Foxp3^+^ regulatory T cell
SSRI =: selective serotonin re-uptake inhibitor
Tconv =: CD4^+^ Foxp3^-^ conventional T cells
Treg =: CD4^+^ Foxp3^+^ regulatory T cell
