# Supplementary material for: Inhibition of acid sphingomyelinase increases regulatory T cells in humans
Source: Brain Commun. 2021 Mar 5;3(2):fcab020. doi: 10.1093/braincomms/fcab020 (PMC8054263; doi:10.1093/braincomms/fcab020)
Supplement: fcab020_Supplementary_Data [file fcab020_supplementary_data.pdf]

Supplemental Data

A

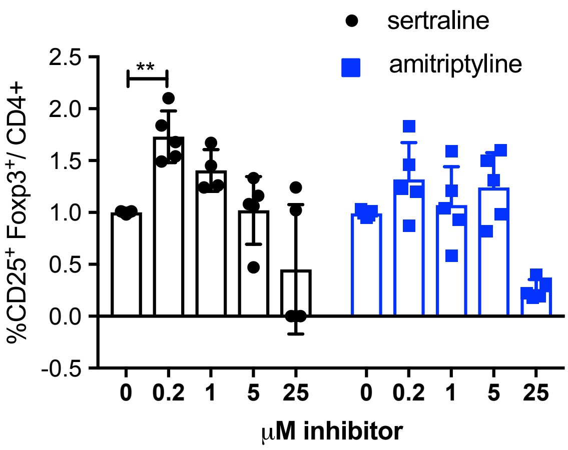

B

	Sertraline	Amitriptyline
Donor 1	0.2 µM	5 µM
Donor 2	0.2 µM	0.2 µM
Donor 3	0.2 µM	5 µM
Donor 4	0.2 µM	5 µM
Donor 5	0.2 µM	0.2 µM

C

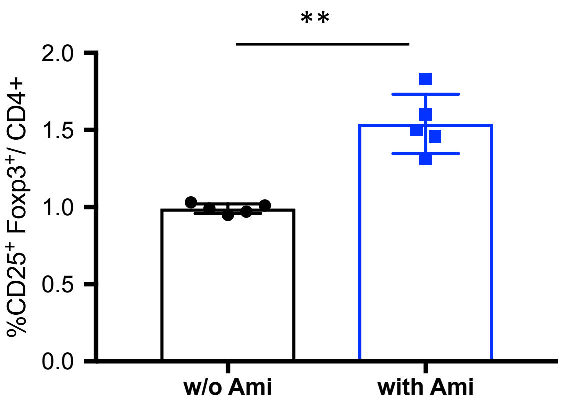

Supplemental Fig. 1. Comparison of sertraline and amitriptyline regarding increase in the proportion of (ef)Treg among CD4$^+$ T cells *in vitro*. Human PBMC of n=5 human donors were cultured as outlined for Fig. 1. (A) Sertraline (black) and amitriptyline (blue) were compared for their effect on efTreg (top 1% regarding CD25 and Foxp3 expression as outlined for Fig. 6). Two-way ANOVA and Tukey post test. (B) Concentrations of sertraline and amitriptyline required to reach optimal increases in the proportion of Treg among CD4$^+$ T cells. (C) Comparison of %efTreg/ CD4$^+$ T cells in the absence (black) and in the presence of optimal concentrations of amitriptyline (blue). Paired two-tailed t-test. **p<0.01.

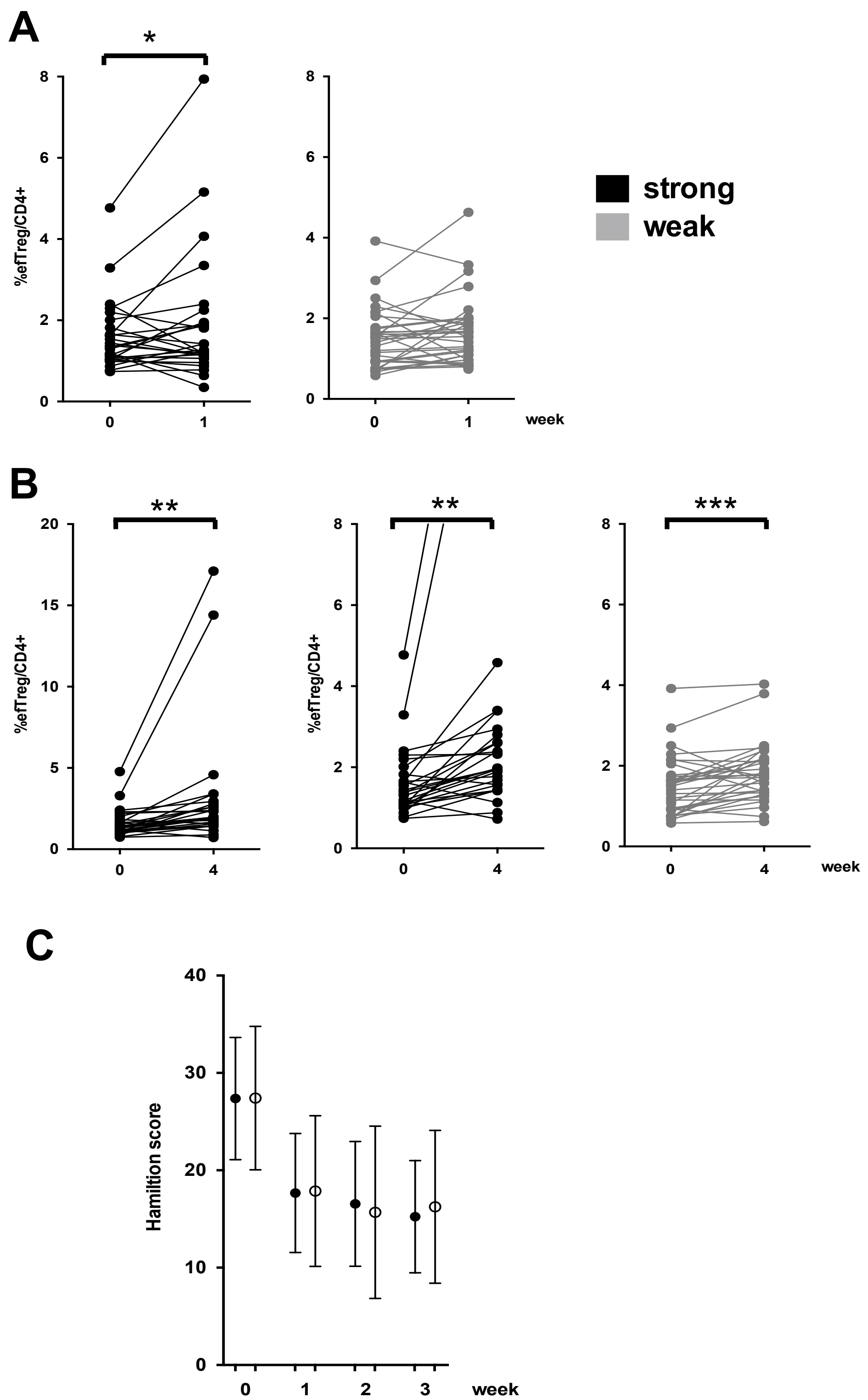

Supplemental Fig. 2. Increase in efTreg frequency among CD4^{+} T cells after treatment of patients and clinical response. (A) and (B) show the same data depicted as group means plus standard deviation in Fig. 3B for individual patients (timepoint zero and after four weeks of treatment). (B) Hamilton Depression Rating Scale (HAMD) scores over the four weeks of hospitalization plotted against the treatment with strong vs. weak ASM-inhibiting antidepressants. A repeated measures analysis of variance (ANOVA) did not reveal significant main or interaction effects of the two groups and the HAMD scores.

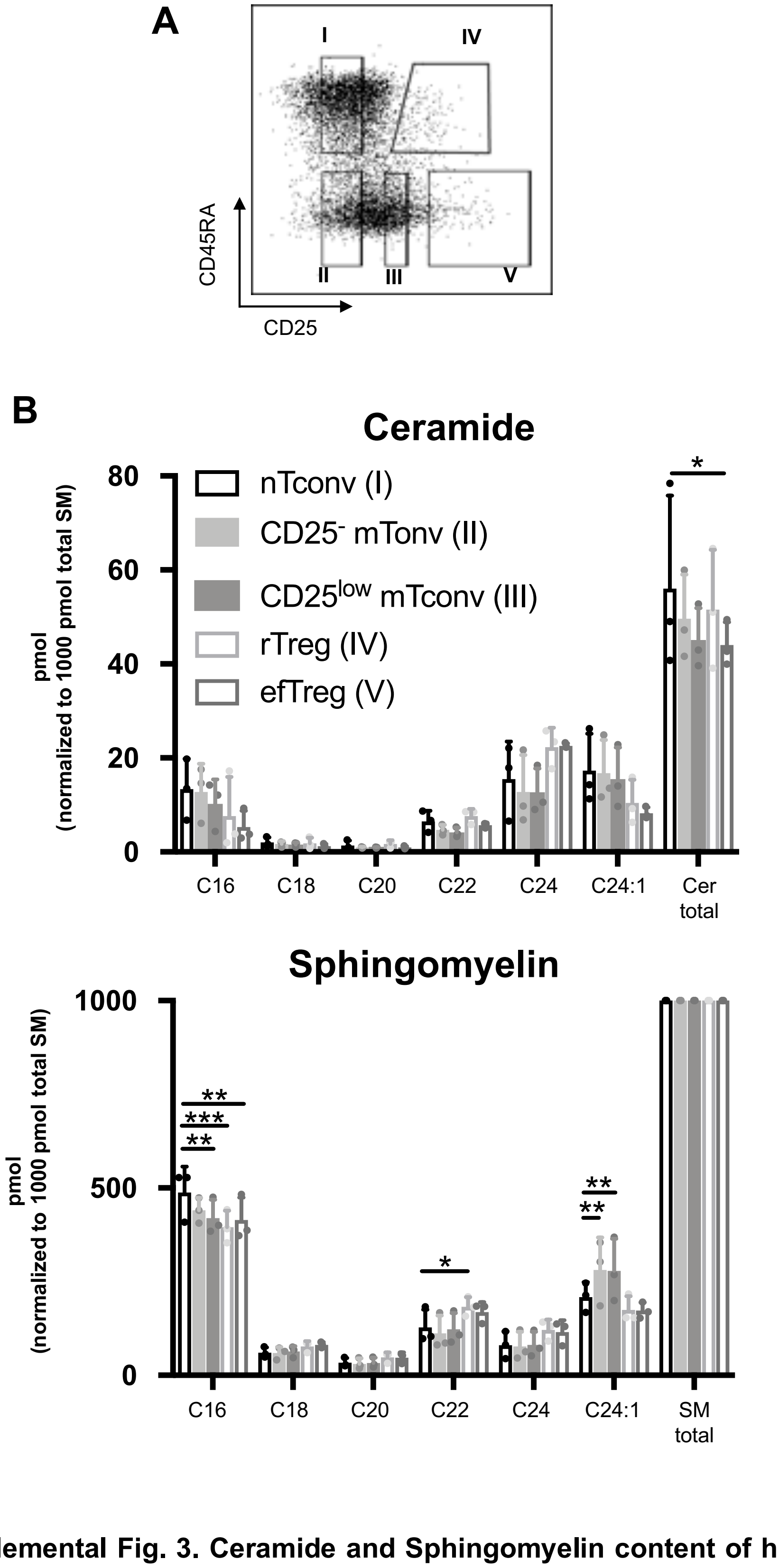

Supplemental Fig. 3. Ceramide and Sphingomyelin content of human Treg and Tconv subsets. (A) Treg and Tconv subsets were sorted as indicated and (B) sphingolipid content determined by mass spectrometry. n = 3 individual experiments/ donors. Individual values and means ± SD are shown. Two-way ANOVA with Tukey post hoc test. * $p < 0.05$, ** $p < 0.01$, *** $p < 0.001$.

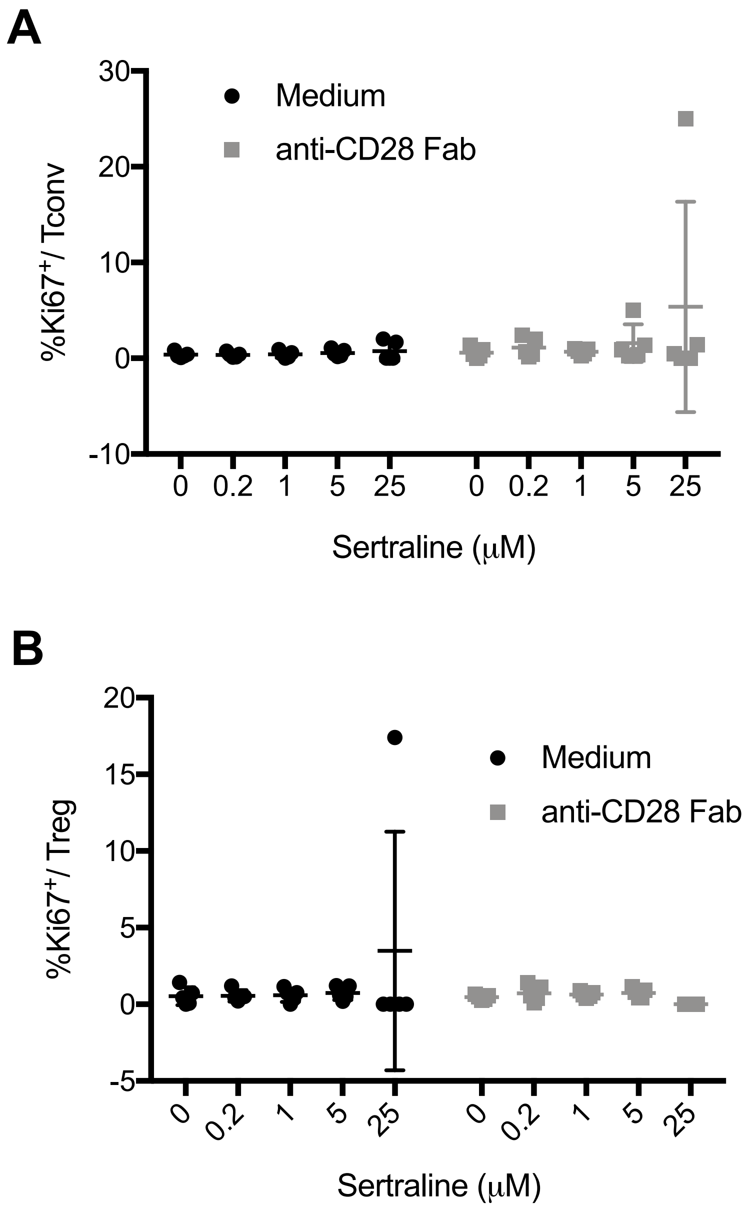

Supplemental Fig. 4. Prolifertation of Treg and Tconv in the presence of anti-CD28 Fab and/ or sertraline. Human PBMC of n=5 different donors were cultured as described for Fig. 6. (A) The proportion of proliferating Ki-67$^+$ cells was determined among Tconv and (B) Treg after two days of high-density culture and four days of culture in the absence (black) or presence (grey) of anti-CD28 Fab and increasing concentrations of sertraline as indicated. Individual values and means ± SD are shown.

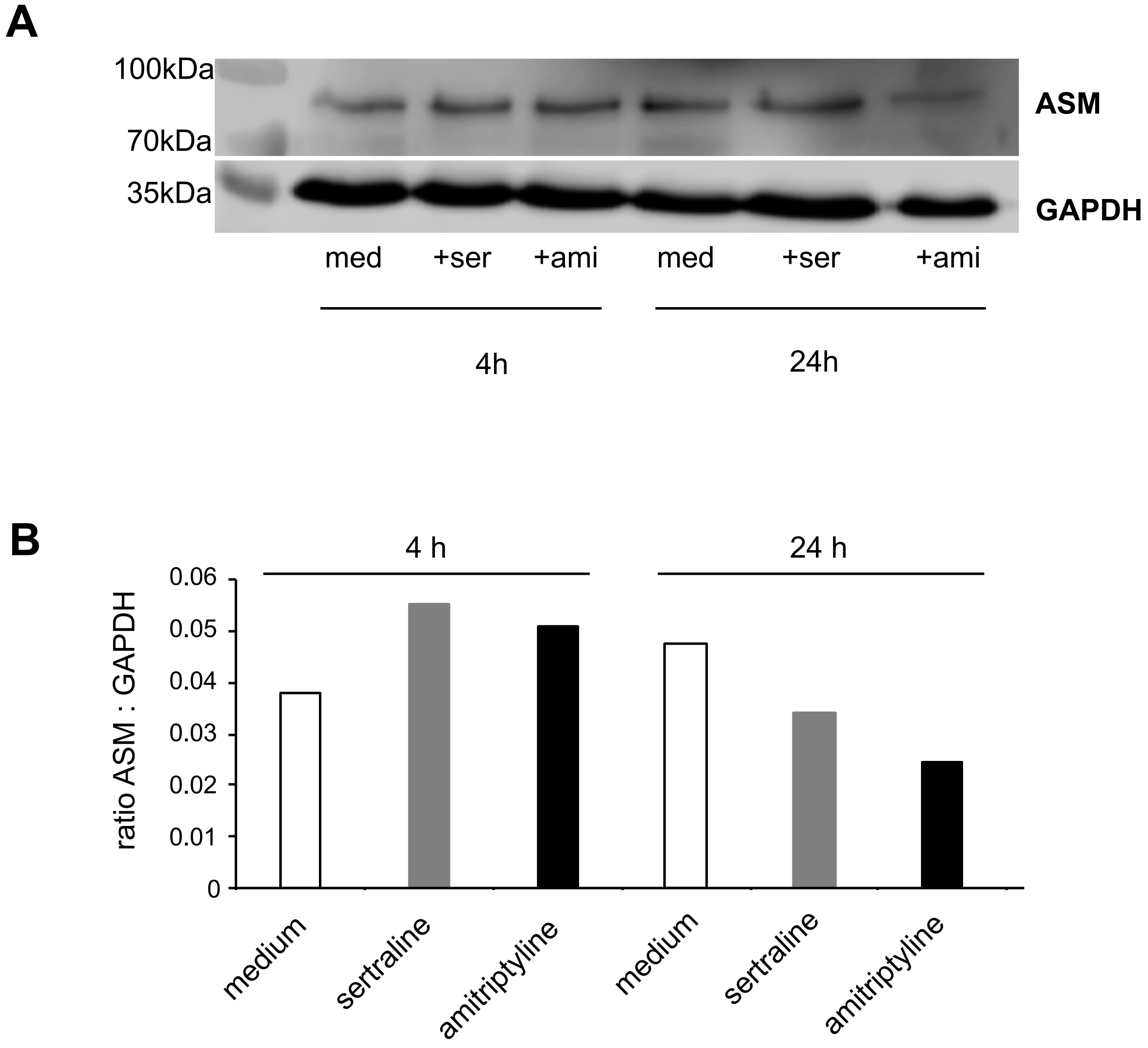

Supplemental Fig. 5. Expression of ASM protein by human CD4$^+$ T cells after culture in the presence of sertraline or amitriptyline. (A) Purified human CD4$^+$ T cells were cultured for 4 or 24 h in the absence or presence of 5 μM sertraline (ser) or amitriptyline (ami). ASM and GAPDH protein expression were determined on Western blots of lysates of the cultured cells. (B) Quantification of ASM protein amount relative to the GAPDH loading control. The experiment was repeated at least once with similar result.

Supplemental Methods

Sphingolipid quantification by HPLC-MS/MS

Cells were resuspended in 500 µL methanol and subsequently subjected to lipid extraction using 1.5 mL methanol/chloroform (2:1, *v:v*) as described (Gulbins *et al.*, 2018). The extraction solvent contained d_7-sphingosine (d_7-Sph), d_7-sphingosine 1-phosphate (d_7-S1P), C17-ceramide (C17 Cer) and C16-d_{31}-sphingomyelin (C16 d_{31}-SM) (all Avanti Polar Lipids, Alabaster, USA) as internal standards. Chromatographic separations were achieved on a 1260 Infinity HPLC (Agilent Technologies, Waldbronn, Germany) equipped with a Poroshell 120 EC-C8 column (3.0 × 150 mm, 2.7 µm; Agilent Technologies). MS/MS analyses were carried out using a 6490 triple-quadrupole mass spectrometer (Agilent Technologies) operating in the positive electrospray ionization mode (ESI+). The following mass transitions were recorded (collision energies (CE) in parentheses): *long-chain bases*: *m/z* 300.3 → 282.3 for Sph (8 eV), *m/z* 307.3 → 289.3 for d_7-Sph (8 eV), *m/z* 380.3 → 264.3 for S1P (16 eV) and *m/z* 387.3 → 271.3 for d_7-S1P (16 eV); *ceramides* (CE = 25 eV for all transitions): *m/z* 520.5 → 264.3 for C16 Cer, *m/z* 534.5 → 264.3 for C17 Cer, *m/z* 548.5 → 264.3 for C18 Cer, *m/z* 576.6 → 264.3 for C20 Cer, *m/z* 604.6 → 264.3 for C22 Cer, *m/z* 630.6 → 264.3 for C24:1 Cer and *m/z* 632.6 → 264.3 for C24 Cer; *sphingomyelins* (CE = 25 eV for all transitions): *m/z* 703.6 → 184.1 for C16 SM, *m/z* 731.6 → 184.1 for C18 SM, *m/z* 734.8 → 184.1 for C16 d_{31}-SM, *m/z* 759.6 → 184.1 for C20 SM, *m/z* 787.7 → 184.1 for C22 SM, *m/z* 813.7 → 184.1 for C24:1 SM and *m/z* 815.7 → 184.1 for C24 SM. Quantification was performed with MassHunter Software (Agilent Technologies).

Comparison of the impact of sertraline and amitriptyline on the proportion of efTreg/ CD4⁺ T cells in vitro

PBMC were isolated and cultured at high density for two days (1 x 10^7 cells/ ml in 1.5 ml/ well of a 24-well flat-bottom-plate; Greiner). Cells were further cultured at a concentration of 1 x 10^6 cells/ml for four days in the presence of titrated concentrations of the ASM inhibitors sertraline or amitriptyline (0, 1, 5, 25 µM). CD25, Foxp3 and CD4 expression were determined by flow cytometry.

Modulation of ASM protein expression in human CD4⁺ T cells by sertraline and amitriptyline

Purified human CD4⁺ T cells (2 x 10^6 cells/ ml) were cultured for 4 and 24 h without or with 5 µM of sertraline or amitriptyline, respectively. T cells were lysed in Western Blot

Lysis buffer (5 M NaCl, 1 M Hepes/Tris , 240 mM ß-Glycerophosphate, 1 M NaF) for 1 h at 4° C and protein concentrations were measured by BCA assay. Protein lysates were mixed with Laemmli buffer and boiled for 1 min prior to SDS-PAGE using a 10% gel. ASM expression was detected with goat-anti-human ASM antibody (R&D) (Naser *et al.*, 2020). To control for protein loading we detected GAPDH expression by mouse anti-rabbit GAPDH (Santa Cruz). Bound antibodies were detected with anti-goat Ig HRP or anti-rabbit Ig HRP, respectively (Jackson Immuno Research) and visualized using ECL substrate (SuperSignal West Pico Plus). Luminescence was detected on the Odyssey® CLx Imaging System (LI COR) and quantified with the Image Studio™ software, version 5.2 (LI COR).

Supplemental References

Gulbins A, Schumacher F, Becker KA, Wilker B, Soddemann M, Boldrin F*, et al.* Antidepressants act by inducing autophagy controlled by sphingomyelin-ceramide. Mol Psychiatry 2018; 23(12): 2324-46.

Naser E, Kadow S, Schumacher F, Mohamed ZH, Kappe C, Hessler G*, et al.* Characterization of the small molecule ARC39, a direct and specific inhibitor of acid sphingomyelinase in vitro. J Lipid Res 2020; 61(6): 896-910.
